# Dominant odor-active events link plant volatile signals to human perception and behavioral intentions in urban smellscapes

**DOI:** 10.3389/fpls.2026.1884543

**Published:** 2026-07-17

**Authors:** Yuetong Yan, Xiujun Wang, Lei Gong

**Affiliations:** School of Landscape Architecture, Beijing Forestry University, Beijing, China

**Keywords:** approach-avoidance, OAV, odorants, olfaction, smellscape, smellwalk, VOCs

## Abstract

**Introduction:**

Urban smellscapes are important components of multisensory green-space experiences, yet quantitative links between plant volatile signals, odor perception, and behavioral responses remain poorly understood.

**Methods:**

We developed an olfactory event–based framework integrating GC–MS volatile profiling, odor activity value (OAV) analysis, ambient-air relative OAV indices, spatial–temporal attenuation assessment, semantic differential evaluation, and behavioral-intention modeling. Six ornamental species with contrasting odor profiles were analyzed to characterize chemical signals, perceptual responses, and stop–avoid intentions.

**Results:**

Perceived olfactory signatures were better explained by temporally dynamic subsets of dominant high-OAV compounds than by total volatile emissions. Dominant odor-active compounds exhibited species-specific emission rhythms and distance-dependent attenuation, defining distinct perceptible odor ranges across vegetation contexts. Semantic evaluation revealed that plant identity and odor recognizability primarily structured olfactory perception, while vegetation context influenced perceived intensity and affective responses. Cross-validated logistic regression identified two behavioral pathways: stop intention was associated with favorable semantic attributes and dominant OAV/relative OAV-based exposure indices, whereas avoid intention was associated with negative odor valence and complex background conditions.

**Discussion:**

These findings indicate that urban plant odors operate as discrete olfactory events shaped by chemical dominance, spatial persistence, perceptual interpretation, and contextual modulation. The proposed framework links odor-active compounds, atmospheric dispersion, human perception, and behavioral intention, providing a quantitative basis for odor-informed urban green-space design.

## 1 Introduction

Urban and near-natural landscapes are increasingly understood as multisensory environmental systems rather than solely visual or ecological assemblages. Among sensory modalities, olfaction is particularly relevant because odor cues are closely linked to emotion- and memory-related processes (Herz, 2026), and recent evidence suggests that plant-rich scentscapes may contribute to measurable health benefits (Tucker et al., 2026). In urban green spaces, plant-derived volatile organic compounds (VOCs), including terpenoids, phenylpropanoids, fatty acid derivatives, and sulfur- or nitrogen-containing compounds, constitute a major chemical basis of olfactory experience (Dudareva et al., 2013). Although these compounds have been extensively studied as ecological signals mediating pollination, defense, and stress responses, they can also function as chemical signals that engage animal and human chemosensory systems (Bouwmeester et al., 2019; Taufer et al., 2025). Analogous to the soundscape concept, smellscape research emphasizes that odors have spatial structure, temporal variability, and place-specific meanings (Henshaw, 2013). Importantly, however, a smellscape should not be understood merely as the physical distribution of odorants, but as a situated sensory experience shaped by chemical exposure, perceptual interpretation, cultural memory, and spatial context. Recent studies have begun to quantify plant-derived scentscapes in urban green spaces (Kay et al., 2026), but most urban olfactory research still lacks mechanistic integration of chemical exposure, perceptual evaluation, and behavioral response (Wang et al., 2024).

A key challenge is that odor perception cannot be inferred directly from total VOC emission. Floral scents are often characterized by strong species specificity and diel rhythmicity (Dudareva and Pichersky, 2006), but human aroma perception is frequently dominated by a small subset of low-threshold odor-active compounds (Tsumura et al., 2023; Ren et al., 2025). The odor activity value (OAV), which relates compound concentration to human olfactory threshold, provides a quantitative metric for identifying molecules with disproportionate perceptual relevance. Because perceived odor depends jointly on chemical abundance and detection threshold, OAV can translate plant volatile profiles into biologically interpretable exposure metrics; nevertheless, it remains rarely incorporated into spatial ecological or behavioral models of urban green spaces. A further limitation concerns the spatial and psychological transformation of odor signals. In open landscapes, plant volatiles attenuate with distance and are modified by vegetation configuration, airflow, and background odor complexity. Although atmospheric dispersion theory provides a physical basis for describing concentration decay (Pasquill and Smith, 1983a), few studies have coupled spatial attenuation with perceptual thresholds to define perceptual odor boundaries, namely the spatial ranges within which odor-active compounds remain perceptually relevant (Mainland et al., 2014). At the same time, the affect-mediated approach–avoidance model proposes that environmental stimuli influence behavior through affective dimensions such as pleasure, arousal, and dominance rather than through physical intensity alone (Xu et al., 2026; Nasar, 1992). This implies that approach and avoidance responses to plant odors may depend not only on exposure magnitude, but also on odor recognizability, pleasantness, and contextual interpretation. Yet integrated models linking threshold-weighted chemical exposure, semantic perception, and spatial behavior remain scarce, hindering statistically grounded inference between olfactory exposure and behavioral response (Dalton, 2000; Majid and Burenhult, 2014).

To address these gaps, this study develops and empirically tests a cross-scale framework linking plant volatile chemistry, odor-active exposure, perceptual semantics, and approach–avoidance behavior in urban green-space contexts. Using six representative aromatic ornamental species, we combined GC–MS-based volatile profiling, OAV-weighted exposure assessment, spatial attenuation analysis, semantic differential evaluation, and behavioral response modeling. Specifically, we asked whether perceived odor signatures are better explained by dominant odor-active compounds than by total VOC emission, how temporal emission patterns and spatial attenuation shape perceptible odor ranges, and how chemical exposure and semantic valence jointly predict stop and avoid intentions. By integrating dominant odor-active molecules, temporal emission windows, perceptual boundaries, and behavioral probabilities, this study provides design-relevant parameters for plant selection, spatial configuration, and visitor-experience planning in urban green spaces.

## 2 Materials and methods

### 2.1 Plant materials and experimental design

**Figure 1:**
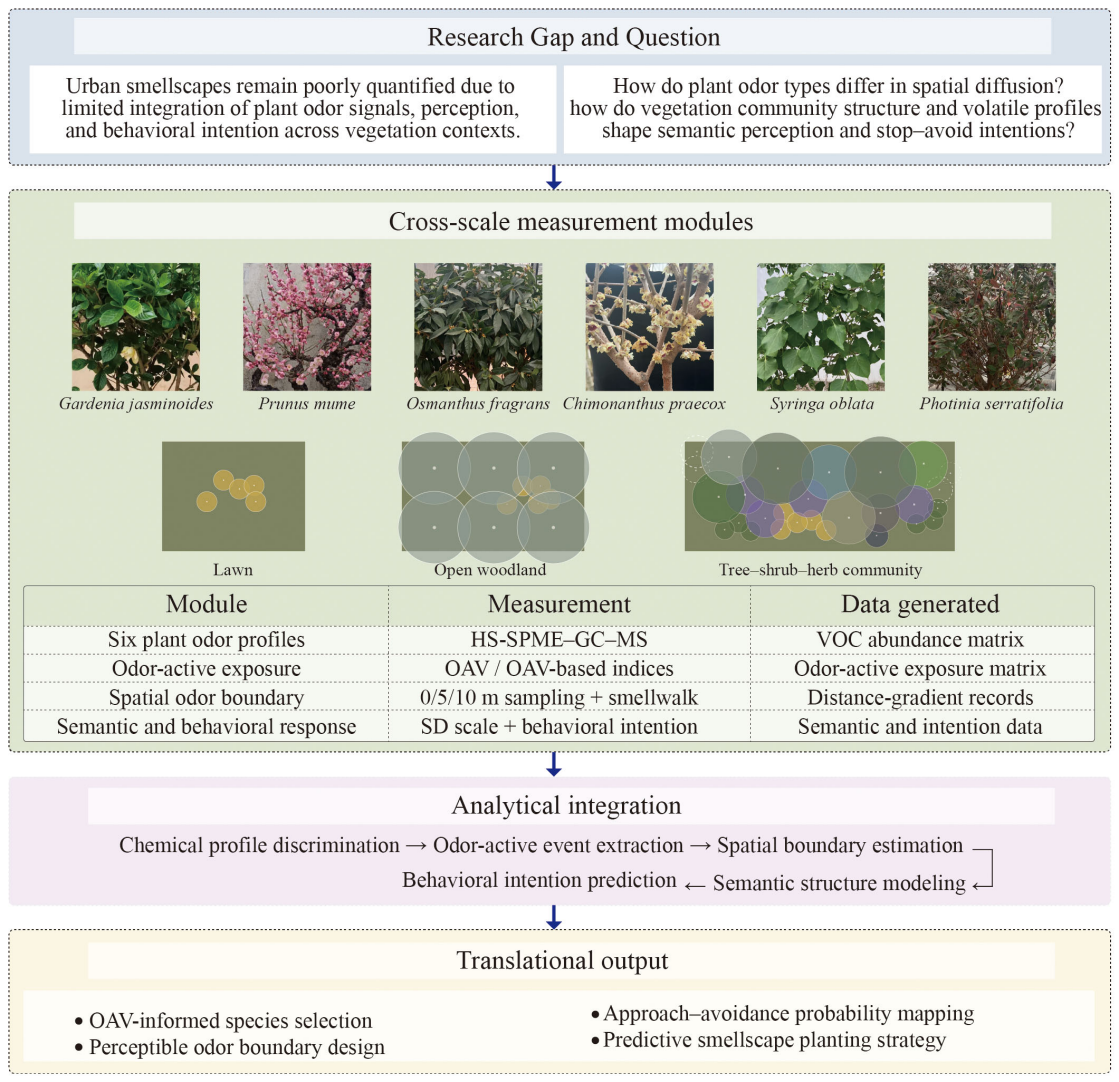
Cross-scale experimental framework linking plant odor signals, vegetation context, semantic perception, and behavioral intention in urban smellscapes.

Five representative ornamental fragrant plant species and one malodorous plant species were selected as the research subjects: wintersweet (*Chimonanthus praecox*, Cp), Osmanthus (*Osmanthus fragrans*, Of), Mei (*Prunus mume*, Pm), Lilac (*Syringa oblata*, So), Gardenia (*Gardenia jasminoides*, Gj), and Photinia (*Photinia serratifolia*, Ps) (**Table 1**). The overall study framework, including plant odor profiling, spatial diffusion measurement, semantic–behavioral evaluation, and translational analysis, is summarized in **Figure 1**. For each species, five healthy potted individuals with comparable growth status and no visible pest or disease symptoms were prepared.

**Table 1:** Morphological characteristics and sampling conditions of experimental plants.

Sample	Cultivation	Height (m)	Crown width (m)	Flowering stage	Sampling temperature (°C)	Number
Cp	container-grown plants	1.73 ± 0.26	0.73 ± 0.14	Full bloom	3	5
Of	container-grown plants	2.56 ± 0.62	1.26 ± 0.31	Full bloom	20	5
Pm	container-grown plants	2.19 ± 0.54	0.92 ± 0.24	Full bloom	11	5
So	container-grown plants	1.33 ± 0.19	1.09 ± 0.22	Full bloom	15	5
Ps	container-grown plants	2.85 ± 0.33	1.13 ± 0.30	Full bloom	14	5
Gj	container-grown plants	0.93 ± 0.12	0.69 ± 0.19	Full bloom	25	5

To characterize diel rhythmicity and temporal dynamics of floral volatile emission, repeated sampling was conducted for each target species during its respective peak flowering period at three standardized time points, 06:00, 13:00, and 19:00. Because the flowering periods differed among species, sampling was carried out at the peak bloom stage of each species rather than on the same calendar date. For each species and time point, flowers were collected from three independent individuals as biological replicates. To quantify spatial attenuation patterns of odor signals, a spatial gradient experiment was conducted within the same lawn setting. For each species, peak-flowering potted individuals were placed in the lawn during their corresponding flowering period, and synchronous sampling was performed at three distances, 0 m, 5 m, and 10 m, from the representative odor source. Volatile samples collected along this distance gradient were used to evaluate the spatial diffusion and attenuation characteristics of plant-derived odor signals. All measurements were conducted under meteorological conditions characterized by minimal wind speed to reduce variability attributable to atmospheric turbulence; detailed information on other weather conditions is provided in **Supplementary Table 1**.

### 2.2 Collection of floral volatile compounds

Floral volatiles were enriched using headspace solid-phase microextraction coupled with gas chromatography–mass spectrometry (HS-SPME–GC–MS) to characterize floral volatile composition and assess the relative spatial attenuation of odor exposure. Two complementary sampling modules were implemented. The first module involved in-vial floral headspace sampling, which was used to characterize interspecific volatile composition, chemical class distribution, and diel dynamics of key compounds. The second module involved spatial air sampling, in which ambient air volatiles were collected at defined distances from the floral odor source to evaluate relative source-to-ambient odor signal attenuation.

For the in-vial floral headspace module, fresh flowers, 1.5–2.0 g per sample, were placed into 10 mL headspace vials. The vials were rinsed, ethanol-washed, oven-dried, and weighed before use. Methyl laurate, prepared as a 10^−4^ mg·mL^−1^ solution, was used as the internal standard, and 2 μL of the internal standard was added to each vial using a 10 μL microsyringe at a fixed position. The vials were immediately sealed, and volatiles were extracted at 25 ± 2 °C for 30 min using a 50/30 μm DVB/CAR/PDMS SPME fiber, which was conditioned at 250 °C for 3 min before use. For the spatial air-sampling module, peak-flowering potted individuals of each target species were placed within the same lawn setting during their respective peak flowering periods. The 0 m samples represented source-level floral headspace samples collected using the in-vial workflow described above, whereas the 5 m and 10 m samples represented ambient air volatile samples collected directly at the corresponding distances from the odor source using gas sampling bags. After collection, gas-bag samples were immediately sealed, protected from direct sunlight, transported to the laboratory in insulated containers at ambient temperature, and analyzed by HS-SPME–GC–MS within the same day. For the ambient-air sampling module, gas-bag blanks were analyzed to evaluate potential background volatile signals derived from the sampling bags and the gas-sampling procedure. For the ambient air samples, the same internal standard solution was added to each gas sampling bag before extraction to allow semi-quantitative correction. Volatile adsorption was then conducted from the gas sampling bags using the same SPME fiber type and extraction duration, 30 min, as used for the in-vial floral headspace samples, and all samples were analyzed using the same GC–MS procedure. This design was used to evaluate the relative attenuation of plant-derived odor signals from the floral source to the surrounding air rather than to establish an absolute concentration decay curve. Each species × time-point combination in the floral headspace module included three biological replicates, and each species × time-point × distance combination in the spatial gradient module was sampled in triplicate.

GC–MS analysis was performed using a DB-5MS fused-silica capillary column, 30 m × 0.25 mm × 0.25 μm. Helium, 99.999%, was used as the carrier gas. The injector temperature was set at 250 °C, and samples were analyzed in split mode with a total flow rate of 27.2 mL·min^−1^ and a split ratio of 20:1. The oven temperature program was as follows: 50 °C for 1 min, ramped at 5 °C·min^−1^ to 120 °C, then at 8 °C·min^−1^ to 200 °C, and finally at 12 °C·min^−1^ to 250 °C, where it was held for 7 min. The total run time was 36.17 min. The ion source temperature was maintained at 200 °C and the interface temperature at 250 °C. Mass spectrometric detection was conducted in full-scan mode with a detector voltage of 1 kV and a scan range of m/z 30–500.

### 2.3 Volatile identification, semi-quantification, and OAV/relative OAV-based index calculation

Compounds were identified or annotated by matching their mass spectra against the NIST library, together with retention time, diagnostic ions, manual spectral interpretation, and, where available, retention-index information from the literature or database records. Authentic standards were used for a subset of representative compounds, including hexyl acetate, eugenol, benzyl acetate, benzyl alcohol, benzaldehyde, cinnamyl acetate, linalool, and indole; all other compounds were annotated without compound-specific authentic standards. Peak areas were automatically integrated and manually verified. Relative abundances for compositional comparisons were calculated by area normalization, and semi-quantification was performed using methyl laurate as the internal standard. For in-vial floral headspace samples, semi-quantified volatile contents were expressed on a fresh-weight basis, ng·g^−1^ FW, and calculated as:


$$Content=\frac{A_{target}}{A_{IS}}\times C_{IS}\times V_{IS}\times 1000÷FW$$

where Atarget and AIS denote the peak areas of the target compound and the internal standard, respectively; CIS is the internal standard concentration, mg·mL^−1^; VIS is the volume of internal standard added, μL; and FW is the fresh weight of the floral sample, g. The factor 1000 was used to convert mg·mL^−1^ × μL to ng.

For ambient air samples, volatiles were collected using pre-cleaned 2 L gas sampling bags. Because these samples did not have a corresponding fresh weight, volatile levels were expressed as internal-standard-normalized semi-quantitative abundances rather than ng·g^−1^ FW. The normalized abundance of each compound was calculated as.


$${\text{Normalized abundance}}_{i}=A_{i}/A_{IS}$$

where A_i_ is the peak area of compound i, and A_IS_ is the peak area of the internal standard.

Odor activity values (OAVs) or OAV-based indices were calculated as:


$$OAV_{i}=C_{i}÷OT_{i}$$

where C_i_ represents the semi-quantified content of compound i in floral headspace samples or the internal-standard-normalized semi-quantitative abundance in ambient air samples, and OT_i_ represents the odor threshold of compound i. Before OAV calculation, compound abundance data and odor threshold values were harmonized to comparable units whenever possible. For ambient air samples, OAV-based indices rather than absolute OAVs were calculated because volatile levels were expressed as internal-standard-normalized semi-quantitative abundances. Threshold values were preferentially obtained from authoritative compilations (Van Gemert, 2011; Devos et al., 1990; Alex et al., 2024) and were standardized to consistent units and matrices. When multiple threshold values were available, values determined under controlled conditions and in a medium most comparable to the sampling context were prioritized. The odor threshold values used in this study are provided in **Supplementary Table 2**. Compounds without reliable odor threshold values were excluded from total OAV calculations for floral headspace samples and from relative OAV-based index calculations for ambient-air samples, but were retained in compositional analyses.

To characterize the dominant odorant-based exposure structure, dominant odor-active compounds were defined as the smallest subset of compounds ranked by descending contribution to the total computable OAV for floral headspace samples or to the total relative OAV-based index for ambient-air samples, cumulatively accounting for 90% of the corresponding total metric within a sample.

### 2.4 Multivariate statistical and profile discrimination analyses

Differences in volatile profiles were explored using principal component analysis (PCA). Orthogonal partial least squares discriminant analysis (OPLS-DA) models were constructed using peak area matrices and OAV/relative OAV-based index attenuation patterns, with model robustness evaluated by cross-validation and permutation tests. Compounds contributing strongly to group discrimination were screened based on variable importance in projection scores, with VIP > 1 used as the primary threshold, and were further interpreted together with their relative abundance and OAV contribution patterns. Volatiles were classified according to chemical structure, including monoterpenes, oxygenated monoterpenes, phenylpropanoids, and aliphatic compounds, and the relative proportions of each class were calculated. Associations between key volatiles and semantic factor scores were assessed using correlation analysis and visualization, whereas species-related differences in volatile profiles were examined using multivariate discrimination and group-wise comparisons. To characterize spatiotemporal odor dynamics, total and dominant OAVs were calculated for floral headspace samples, whereas corresponding total and dominant relative OAV-based indices were calculated for ambient air samples collected at 5 m and 10 m. These metrics were compared across spatial distances, 0, 5, and 10 m, and time points, 06:00, 13:00, and 19:00. Core molecular types maintaining detectable contributions during spatial diffusion were identified based on relative OAV/OAV-based index retention ratios and contribution patterns. All multivariate analyses and visualizations were performed in R, Python, and SIMCA 14.0, with river and Sankey plots generated using OmicShare.

### 2.5 Dynamic odor perception recording

Dynamic odor perception was assessed using the smellwalk method, an *in situ* sensory survey approach widely applied in environmental perception research (Henshaw, 2013; Xiao et al., 2020). For each arranged plant-group scenario, six trained student evaluators with normal olfactory function were recruited. Normal olfactory function was first screened by self-reporting of no olfactory or respiratory disorders and further assessed using a brief four-item odor identification test. Before the survey, evaluators were familiarized with the recording protocol and instructed to avoid smoking, alcohol consumption, strongly flavored food, and scented personal products prior to assessment. Surveys were conducted at 9:00 a.m. during the flowering period under sunny conditions and calm or light-wind conditions. Wind speed was monitored during each survey and kept within a comparable low range. Participants walked along a predefined route starting from a point 30 m outside the arranged plant group, moving toward the group, and then continuing to a point 30 m beyond it. During the walk, evaluators recorded odor type, perceived odor intensity, odor continuity, first detection point, and disappearance point using the same recording protocol. Route length, walking protocol, recording procedure, and survey timing were kept consistent across all measurements to ensure comparability.

### 2.6 Semantic differential assessment across spatial contexts

A semantic differential (SD) scale was used to quantify olfactory landscape perception across four dimensions: perceived intensity and detectability, odor quality, emotional–restorative response, and cognitive recognition (**Table 2**). All items were rated on a symmetric five-point scale from −2 to +2, with higher scores indicating stronger alignment with the positive semantic pole. Internal consistency of each dimension was evaluated using Cronbach’s α. The experiment was conducted in Nanhu Park, Hangzhou. For each of the six target plant species, an independent group of 148 voluntary adult participants was recruited *in situ* from three vegetation contexts: lawn (n = 60), open woodland (n = 48), and tree–shrub–grass community (n = 40), resulting in 888 participants in total. Each participant was assigned to only one plant-by-context condition. Because the flowering periods differed among plant species, each target plant species was evaluated during its corresponding peak flowering period by different participants recruited from the corresponding vegetation context. Each plant species was evaluated in all three vegetation contexts during its corresponding peak flowering period, forming a fully crossed Plant × Space type design. Different participants were recruited for different plant-by-context combinations, and each participant completed only one questionnaire.

**Table 2:** Smellscape perception form.

Dimension	Negative anchor	Rating scale					Positive anchor
Perceived Intensity	Weak	-2	-1	0	1	2	Strong
	Vague	-2	-1	0	1	2	Clear
	Hard to perceive	-2	-1	0	1	2	Easy to perceive
	Fluctuating	-2	-1	0	1	2	Stable and continuous
Odor Quality	Irritating	-2	-1	0	1	2	Gentle
	Simple	-2	-1	0	1	2	Rich
	Chaotic	-2	-1	0	1	2	Harmonious
Emotional and Restorative Response	Tense	-2	-1	0	1	2	Relaxed
	Unpleasant	-2	-1	0	1	2	Pleasant
	Oppressive	-2	-1	0	1	2	Soothing
Cognitive and Identification Attributes	Artificial	-2	-1	0	1	2	Natural
	Hard to identify	-2	-1	0	1	2	Easy to identify

Participants were healthy adults, 38.5 ± 11.0 years, with no reported respiratory or olfactory disorders. Participants reported no smoking, alcohol consumption, strongly flavored food intake, or use of scented products on the day of testing. A static exposure paradigm was adopted. Participants remained quietly for 10 min at a designated exposure point near the arranged plant group before independently completing the questionnaire. In the behavioral-intention assessment conducted alongside semantic evaluation, “stop” was operationally defined as self-reported willingness to remain and observe the arranged plant group, whereas “avoid” was defined as self-reported willingness to leave the community without stopping. Questionnaires with substantial missing responses or logically inconsistent answers were excluded before analysis. Negatively worded items were reverse-coded where necessary so that higher scores consistently represented more positive perception.

### 2.7 Linear models for semantic perception analysis

To examine the effects of plant species and spatial context on olfactory semantic perception, linear models were constructed with three semantic dimensions—Weak–Strong (perceived intensity), Tense–Relaxed (emotional response), and Unpleasant–Pleasant (pleasantness)—as dependent variables. Plant species (Plant), spatial context (Space type: lawn, open woodland, and tree–shrub–grass community), and their interaction term (Plant × Space type) were specified as fixed effects to test whether spatial context modulated plant-specific olfactory perception patterns. Because the design was fully crossed and each participant completed only one questionnaire, observations were treated as independent, and no subject-level random effect was included. Model assumptions were evaluated using residual diagnostics, and fixed effects were tested with Wald χ² tests. Statistical significance was set at p < 0.05.

### 2.8 Behavioral-intention modeling and cross-validation

In the behavioral-intention assessment conducted alongside the static semantic evaluation, “stop” and “avoid” intentions were treated as binary response variables. Logistic regression models were constructed to characterize the statistical associations between olfactory exposure metrics and self-reported behavioral intentions. Core predictors included the dominant odor exposure metric, defined as the log-transformed dominant OAV for floral headspace samples or the log-transformed dominant OAV-based exposure index for ambient air samples using log_10_(x + 1), and contextual variables such as spatial type. Semantic dimension scores derived from the SD scale were included in extended models to evaluate the proposed chemical exposure–semantic perception–behavior association. Predictor sets were kept parsimonious according to the number of positive and negative cases to reduce the risk of overfitting. Internal model performance was evaluated using stratified five-fold cross-validation, which preserved the proportion of response classes in each fold. The ROC–AUC was used as the primary performance metric, and results were reported as mean ± standard error across the five folds.

## 3 Results

### 3.1 Species-specific volatile signatures and diurnal dynamics of floral scent emission

**Figure 2:**
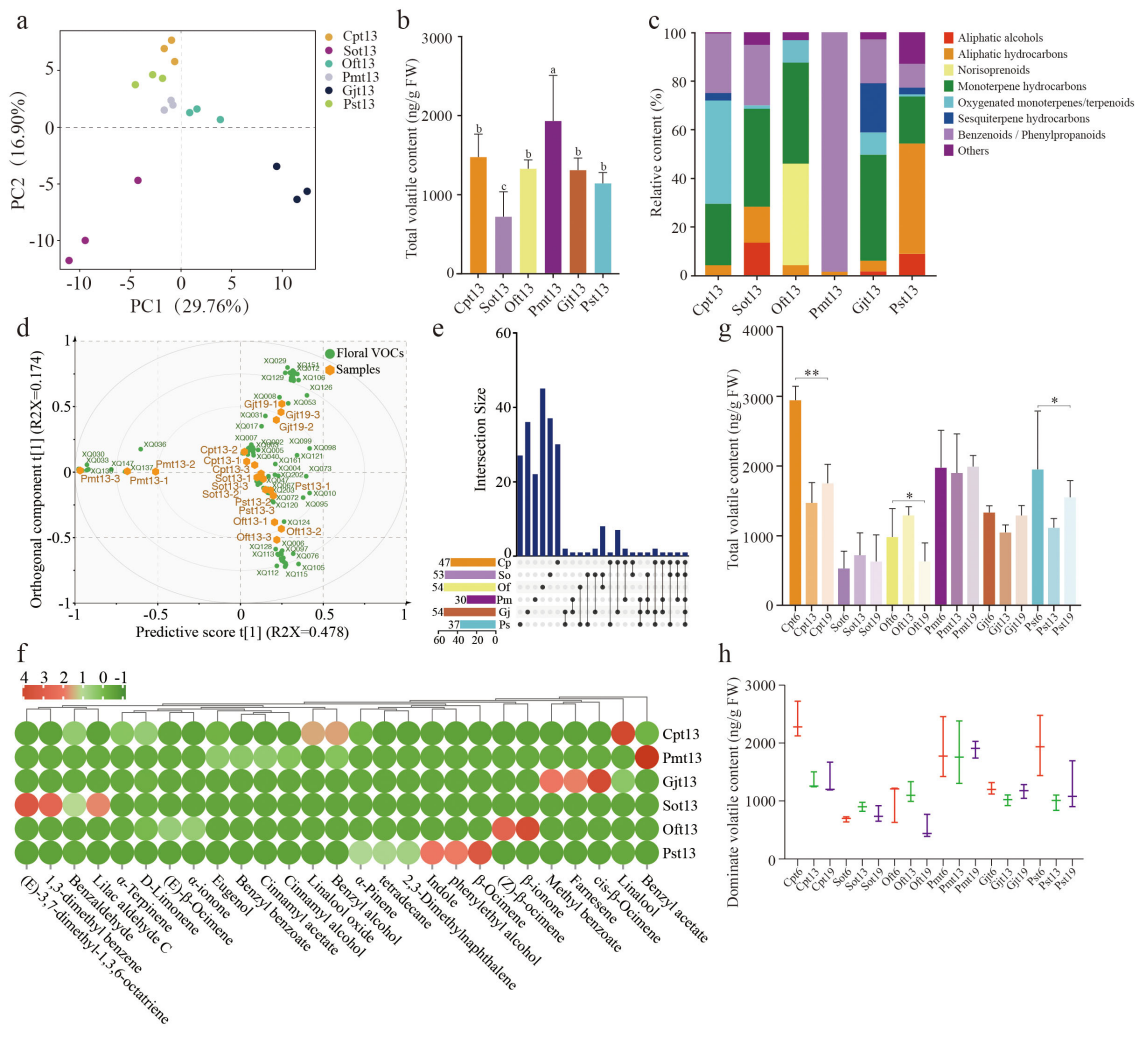
Diurnal variation of floral volatile emission patterns, chemical composition, and key odor-active compounds in six ornamental plant species. **(a)** Principal component analysis (PCA) of floral volatile profiles among the six plant species. **(b)** Total floral volatile content of the six plant species measured at a representative sampling time. **(c)** Relative composition of major chemical classes of floral volatile compounds in each species. **(d)** Orthogonal partial least squares discriminant analysis (OPLS-DA) score plot showing discrimination of floral volatile profiles among species. **(e)** Intersection pattern of floral volatile compounds among six plant species. **(f)** Correlation analysis between key floral volatile compounds and plant species. **(g)** Diurnal variation in total floral volatile content at 06:00, 13:00, and 19:00. **(h)** Diurnal changes in the content of representative odor-active volatile compounds at different time points. Different lowercase letters indicate significant differences among groups based on Tukey’s HSD multiple comparison test (P < 0.05). Asterisks indicate statistical significance (*P* < 0.05; *P < 0.01).

GC–MS profiling revealed pronounced interspecific variation in floral volatile composition among the six ornamental species. PCA separated the species according to their overall volatile profiles, indicating species-specific scent composition (**Figure 2a**). At the representative sampling time point of 13:00, Pm exhibited the highest total volatile content (1936.89 ± 577.27 ng·g^−1^ FW), whereas So showed the lowest level (724.20 ± 315.59 ng·g^−1^ FW); Cp, Of, Gj, and Ps displayed intermediate volatile contents (**Figure 2b**). Chemical class analysis showed distinct compositional patterns (**Figure 2c**): Cp and Of were rich in monoterpene hydrocarbons and oxygenated monoterpenes/terpenoids, which together accounted for 67.97% and 50.64% of their total volatile profiles, respectively; Pm was dominated by benzenoid/phenylpropanoid compounds (98.49%), whereas Ps showed a relatively hydrocarbon-rich profile, with aliphatic and sesquiterpene hydrocarbons together accounting for 31.72%. OPLS-DA further resolved species-specific scent signatures (**Figure 2d**). Cp exhibited an aldehyde–monoterpene composite profile characterized by aromatic aldehydes and oxygenated monoterpenes. So was mainly associated with eugenol and related phenylpropanoids, reflecting a clove-like phenylpropanoid-dominant signature. Of and Gj were characterized by β-ionone and ocimene-type compounds, whereas Pm was enriched in benzenoid compounds such as benzaldehyde and benzyl acetate. In contrast, Ps was distinguished by indole together with selected monoterpenes and sesquiterpenes, forming an indole-dominant pungent scent profile. The number of detected volatile compounds varied among species, ranging from 30 in Pm to 54 in Of, and only a limited subset was shared across all six species (**Figure 2e**). The compound–species association heatmap further visualized species-specific co-variation patterns, with several volatiles clustering preferentially with particular plant species (**Figure 2f**).

Total floral volatile emission exhibited species-specific diurnal patterns across 06:00, 13:00, and 19:00 (**Figure 2g**): Of and Ps showed higher emission at 13:00, Cp was highest at 06:00, whereas Pm and Gj maintained relatively stable emission levels across the three time points. In most species, total emission reached a maximum at 13:00 relative to 06:00 and 19:00, although the magnitude of this midday peak varied among species. The compound–species association heatmap further visualized species-specific volatile patterns (**Figure 2f**). Monoterpene hydrocarbons, including β-ocimene, (E)-β-ocimene, α-pinene, and D-limonene, showed stronger associations with Of and Ps than with Cp and So, whereas benzenoid/phenylpropanoid compounds, such as benzyl acetate, benzyl benzoate, and benzaldehyde, were more closely associated with Pm and Gj. Individual volatiles also showed compound-specific temporal patterns across 06:00, 13:00, and 19:00 (**Figure 2h**), indicating that diurnal scent variation was shaped by both total emission intensity and shifts in specific odor-active compounds (**Supplementary Table 3**).

### 3.2 Odor-active compounds as key predictors of olfactory perception

**Figure 3:**
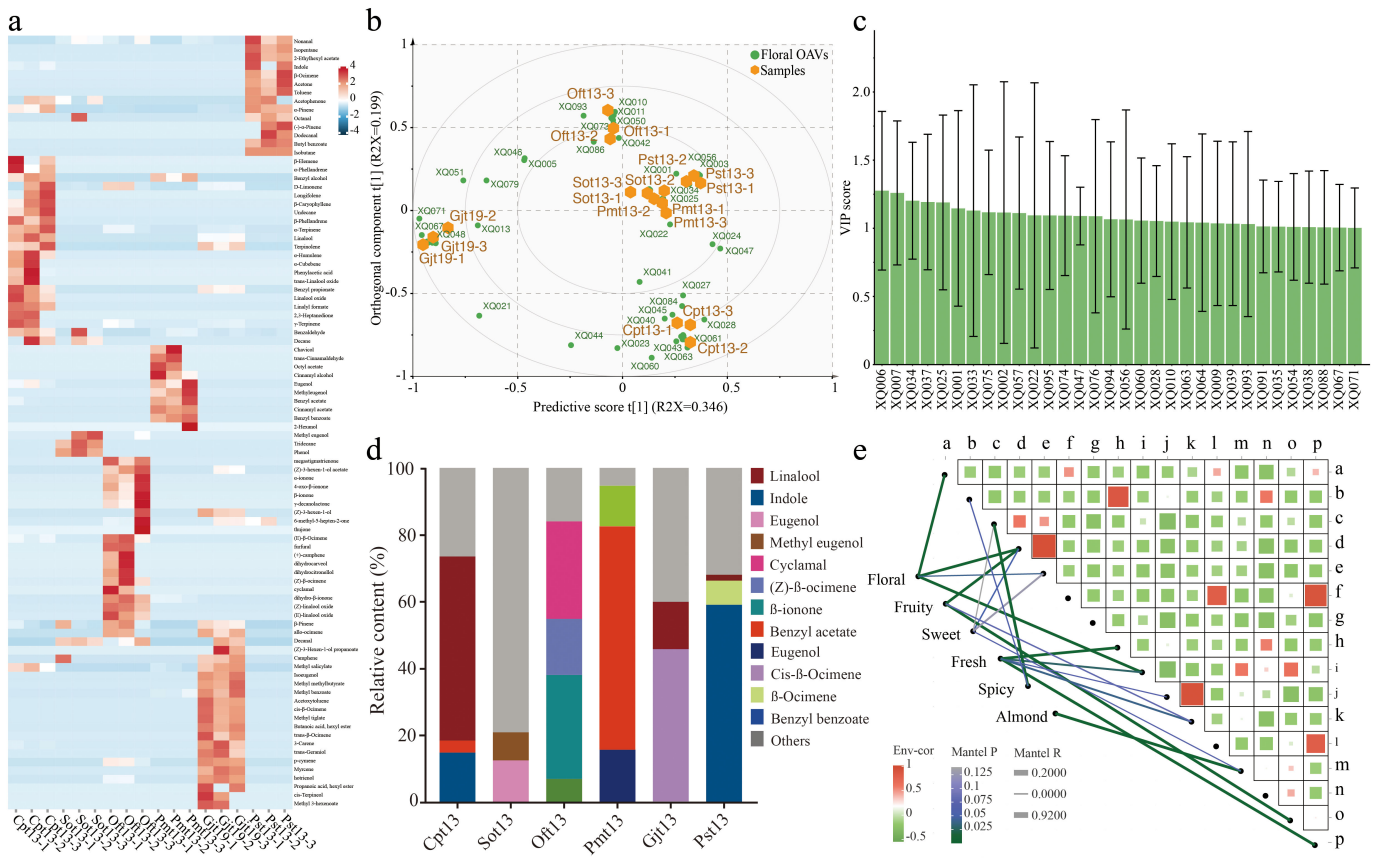
Odor activity value (OAV)-based characterization of floral scent profiles and their sensory relevance across different plant species. **(a)** Heatmap of standardized odor activity values (OAVs) of floral volatile compounds across different plant species. **(b)** OPLS-DA score plot based on OAV-weighted floral volatile profiles. **(c)** Variable importance in projection (VIP) scores derived from the OAV-based OPLS-DA model. **(d)** Relative contribution of major odor-active compounds to the overall floral scent profiles of each plant species, expressed as the proportion of total OAV. **(e)** Correlation analysis between odor activity values of key volatile compounds and sensory perception dimensions.

To identify odor-active compounds associated with interspecific scent differences, OAV-weighted volatile profiles were compared across species. The standardized OAV heatmap showed species-specific enrichment of key odor-active compounds, with Of and Gj characterized by high OAVs of β-ionone, (Z)-β-ocimene, β-ocimene, and benzyl acetate, and So and Ps by selected benzenoid/phenylpropanoid odorants such as eugenol and benzyl benzoate (**Figure 3a**). OPLS-DA further separated floral scent profiles based on OAV-weighted data (**Figure 3b**), and VIP analysis identified β-ionone, (Z)-β-ocimene, benzyl acetate, eugenol, and linalool as major contributors to interspecific discrimination (VIP > 1; **Figure 3c**). Dominant odorant analysis showed that floral scent profiles were largely shaped by a small subset of high-OAV compounds (**Figure 3d**). β-ionone and ocimene derivatives accounted for a large proportion of total OAV in Of and Gj, whereas eugenol-related compounds dominated in So, indicating species-specific reliance on different dominant odor-active compounds. Mantel-based association analysis further indicated that floral, fruity, and sweet semantic attributes were more closely associated with high-OAV compounds, including β-ionone, ocimene-related compounds, and benzyl acetate (**Figure 3e**). Fresh and spicy dimensions were more closely associated with selected monoterpenes and aliphatic aldehydes, whereas the almond attribute showed weak correlations with only a limited number of compounds, suggesting a more restricted contribution to overall scent perception.

### 3.3 Perceptible odor ranges and spatiotemporal attenuation of odor-active exposure

**Figure 4:**
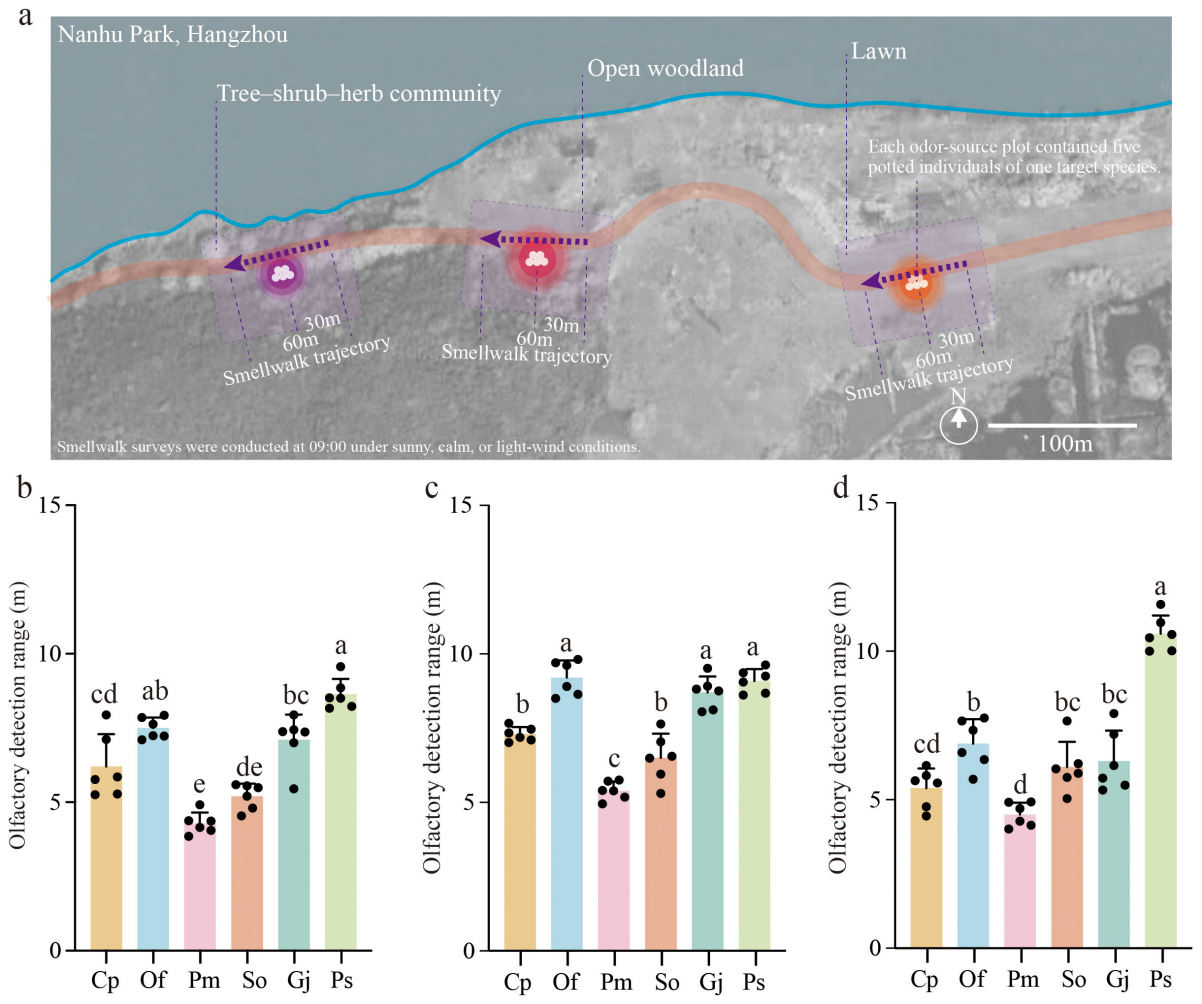
Smellwalk design and perceptible odor ranges of six plant species across three vegetation contexts. **(a)** Schematic layout of smellwalk trajectories in Nanhu Park, Hangzhou. Each odor-source plot contained five potted individuals of one target species. Evaluators walked along a 60 m trajectory from 30 m before the odor source to 30 m beyond it. Smellwalk surveys were conducted at 09:00 under sunny, calm, or light-wind conditions. **(b–d)** Smellwalk-based olfactory detection ranges of six plant species in lawn, open woodland, and tree–shrub–herb community, respectively. Bars represent mean ± SD. Different lowercase letters indicate significant differences among plant species within the same vegetation context based on Tukey’s HSD test at p < 0.05.

Smellwalk records showed clear species- and context-dependent differences in olfactory detection range (**Figure 4a**). In the lawn setting, Ps showed the longest detection range, followed by Of and Gj, whereas Pm showed the shortest range (**Figure 4b**). In the open woodland, detection ranges increased for most aromatic species, with Of, Gj and Ps forming the highest group, while Pm remained the lowest (**Figure 4c**). In the tree–shrub–herb community, Ps exhibited the greatest olfactory detection range and significantly exceeded all other species (p < 0.05; **Figure 4d**).The open woodland provided the most stable context for extending the perceptible range of aromatic plant odors, whereas the tree–shrub–herb community appeared to enhance the persistence of the pungent odor of Ps. These results suggest that vegetation structure modulates olfactory detectability by altering odor retention, dilution, and background odor complexity.

**Figure 5:**
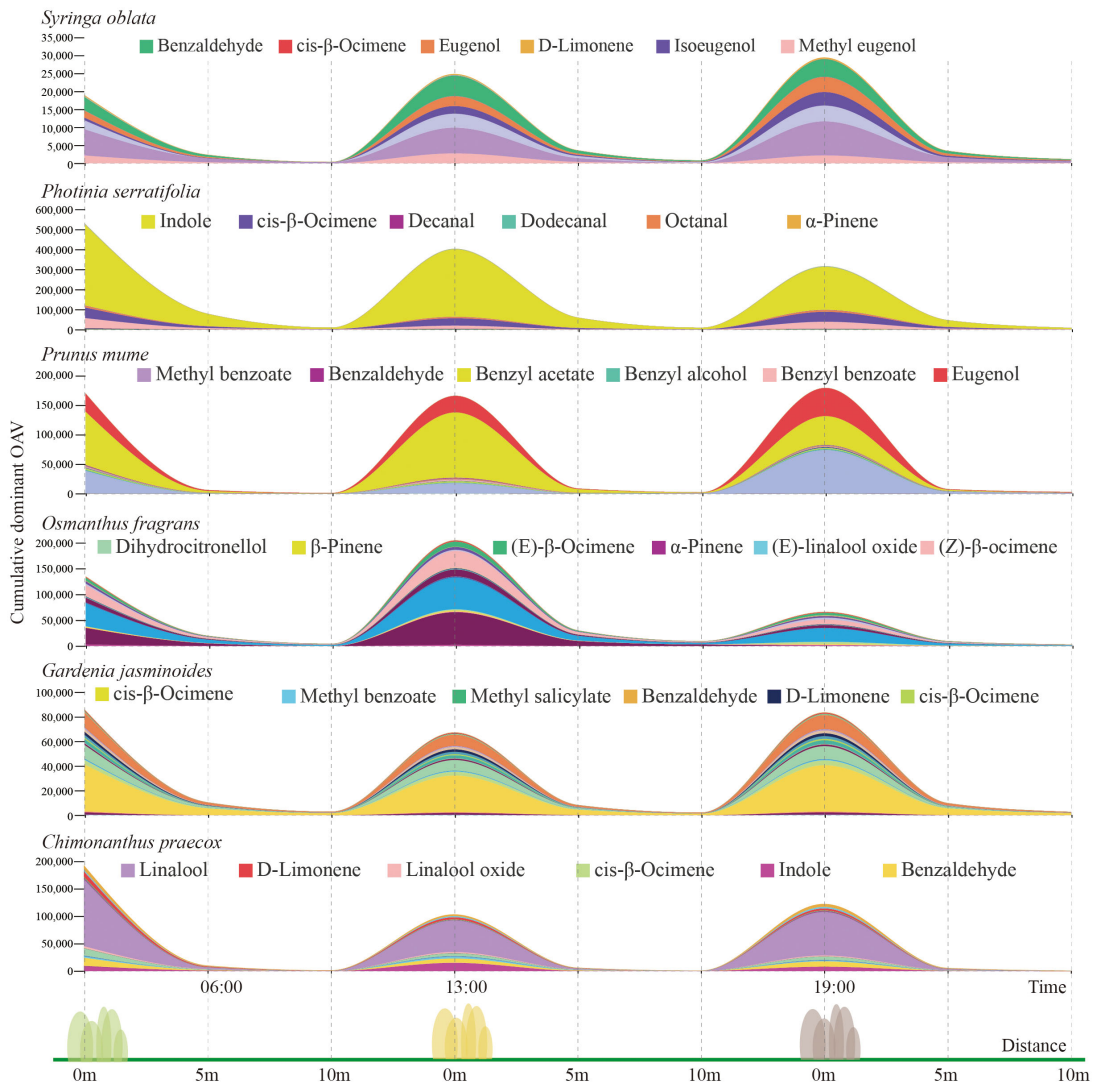
Spatiotemporal restructuring of dominant odorant contributions across plant species. Colors indicate different dominant odorants. The 0 m samples represent source-level floral headspace OAVs, whereas the 5 m and 10 m samples represent relative OAV-based indices for ambient-air samples.

To characterize spatiotemporal odor-active exposure, source-level floral headspace OAVs at 0 m were examined together with relative OAV-based exposure indices for ambient-air samples at 5 m and 10 m across species and sampling times (**Figure 5**). Source-level OAVs were generally highest at 0 m and decreased in the corresponding ambient-air indices at 5 m and 10 m, indicating species- and compound-specific source-to-ambient attenuation rather than an absolute continuous concentration gradient. Peak source-level exposure occurred at different times among species: Cp and Of at 13:00, So and Pm at 19:00, Ps at 06:00, and Gj at 06:00 and 19:00. Dominant odorants also differed in their source-to-ambient persistence. Ps was characterized by indole-related compounds that retained relatively high ambient-air exposure indices at 5 m and/or 10 m, consistent with its larger smellwalk-based detection range. In contrast, Pm showed strong source-level contributions from benzenoid esters, but these compounds contributed less to ambient-air exposure at greater distances, consistent with its shorter detection range. Cp, Of, Gj, and So showed intermediate patterns involving aromatic aldehydes, mono-/oxygenated monoterpenes, ionone derivatives, esters, or phenylpropanoid compounds. Together, these results show that the timing and spatial extent of odor-active exposure differed among species and were associated with differences in dominant odorant composition.

### 3.4 Vegetation context modulates semantic perception of plant-derived odors

Semantic differential ratings varied among plant-derived odor profiles and vegetation contexts, indicating that both plant identity and spatial context influenced perceived intensity, odor quality, emotional response, and recognizability (**Table 3**). In the intensity/detectability dimension, Of, Gj, Cp, and Pm generally received higher scores for strong, clear, and easy-to-perceive odors, whereas Ps and the control showed lower or negative scores across several contexts. For example, Of showed the highest Vague–Clear scores across the three vegetation contexts, ranging from 1.66 to 1.83, whereas Ps remained negative across contexts, ranging from −0.70 to −0.29. Odor quality ratings also differed among species and spatial settings, with floral aromatic species generally receiving more positive ratings for gentleness and harmony than Ps. In the emotional–restorative dimension, Of and Cp tended to receive higher relaxed and pleasant scores, particularly in open woodland–grass settings, whereas Ps was more frequently associated with negative affective ratings. Cognitive and recognition scores also varied with vegetation context, indicating that odor naturalness and recognizability were influenced by both plant odor profile and surrounding spatial setting. Overall, these results show that vegetation context modulated smellscape perception across multiple semantic dimensions.

**Table 3:** Semantic differential ratings of floral odor perception across plant species and vegetation structure types.

Semantic dimension	Cp	So	Of	Pm	Gj	Ps	Ck
Weak – Strong	1.07/1.21/1.43	-0.67/-0.37/-0.28	1.45/1.52/1.78	1.16/1.19/1.48	1.31/1.38/1.58	-0.83/-0.54/-0.55	-1.74/-1.37/-1.08
Vague – Clear	0.97/1.02/1.28	0.08/0.17/0.18	1.66/1.77/1.83	0.58/0.87/0.68	1.08/1.31/1.63	-0.7/-0.29/-0.45	-1.87/-1.48/-1.28
Hard to perceive – Easy to perceive	1.03/1.25/1.35	0.15/0.37/0.3	1.4/1.62/1.65	0.55/0.81/0.95	1.2/1.48/1.45	-0.85/-0.56/-0.53	-1.6/-1.19/-0.95
Fluctuating – Stable	0.88/1.33/1.23	-0.13/0.1/0.08	1.27/1.54/1.48	0.47/0.85/1.28	1.07/1.4/1.38	-0.72/-0.27/-0.48	0.02/0.25/0.38
Irritating – Gentle	1.43/1.56/1.33	1/1.23/0.88	0.85/0.56/0.78	1.2/1.37/1.08	0.4/0.17/0.28	0.1/0.37/0.55	0.4/0.56/0.18
Simple – Rich	0.9/1.1/1.38	0.42/0.46/0.58	1.37/1.46/1.68	0.67/0.75/0.98	1.32/1.46/1.58	-0.63/0.1/-0.18	-1.48/-1.1/-0.63
Chaotic – Harmonious	1.02/1.15/0.98	0.63/0.71/0.55	1.38/1.54/1.45	0.78/1.04/0.65	1.13/1.35/1.25	-1.1/-1.13/-1.28	0.28/0.46/0.05
Tense – Relaxed	1.47/1.48/0.68	0.7/0.92/0.23	1.6/1.67/1.18	1.55/1.48/0.73	1.65/1.73/1.03	-0.48/-0.29/-0.58	0.6/0.87/0.08
Unpleasant – Pleasant	1.17/1.29/1.08	0.67/0.77/0.5	1.47/1.69/1.35	1.17/1.33/1.25	1.27/1.52/1.4	-0.77/-0.46/-0.85	0.42/0.56/0.05
Oppressive – Soothing	1.08/1.27/0.98	1.17/1.02/0.63	1.5/1.6/1.2	1.22/1.33/1.03	1.37/1.52/1.33	-0.9/-1.13/-1.48	0.62/0.94/0.38
Artificial – Natural	1.15/1.54/1.4	0.85/1.27/1.3	1.4/1.65/1.6	1/1.48/1.5	0.95/1.37/1.45	-1.43/-1.63/-1.75	0.7/1.06/1.05
Hard to identify – Easy to identify	0.87/1.15/0.93	-0.08/0.06/0.18	1.65/1.75/1.7	0.77/0.96/0.68	1.47/1.65/1.43	-0.98/-1.23/-1.63	-1.88/-1.46/-1.08

The three slash-separated values correspond to lawn (n = 60), open woodland–grass (n = 48), and tree–shrub–grass (n = 40), respectively. Detailed descriptive statistics and significance comparisons are provided in **Supplementary Table 4**.

### 3.5 Vegetation context modulates species-specific semantic responses to plant-derived odors

**Figure 6:**
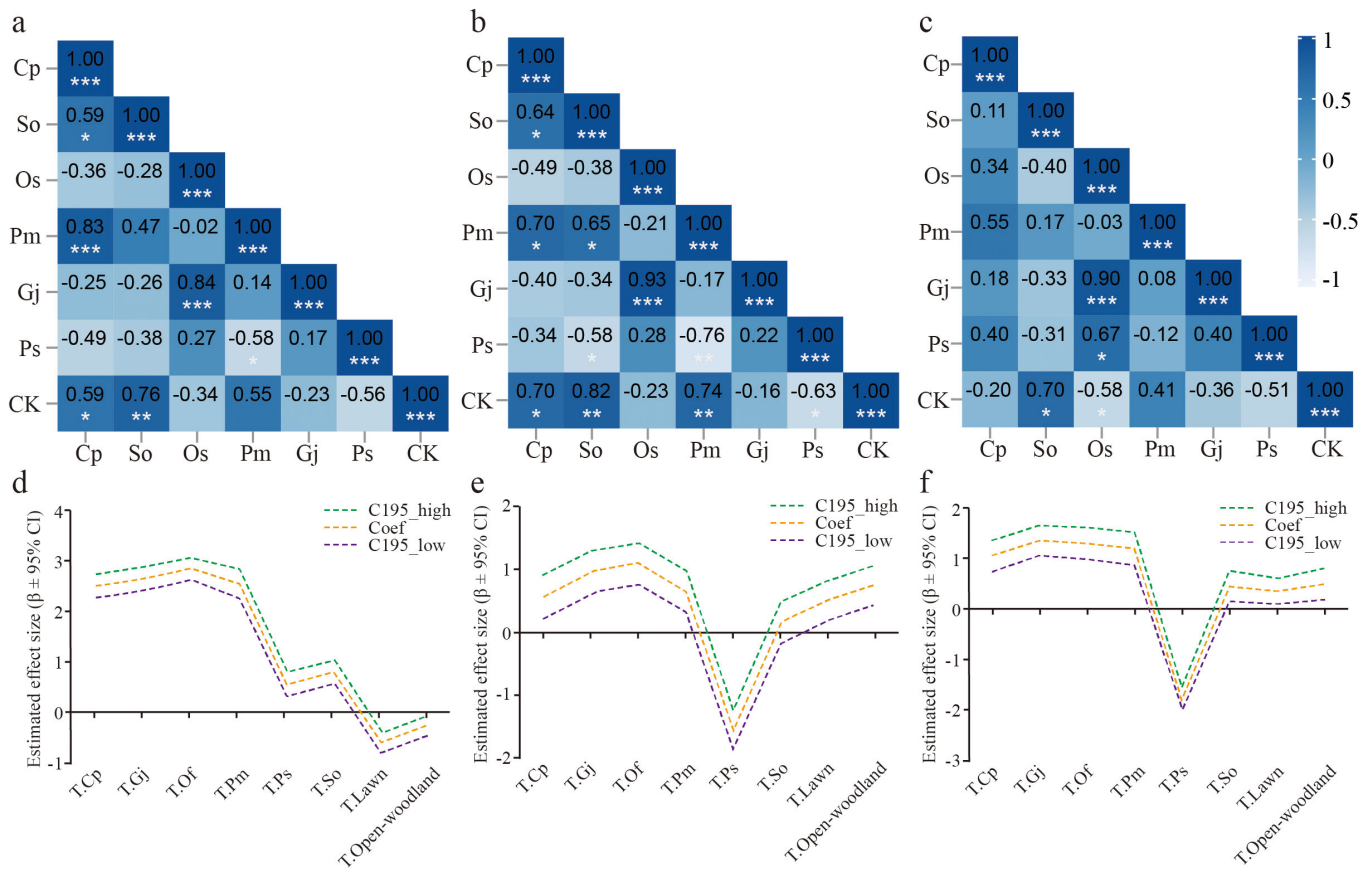
Spatial context modulates interspecific odor perception structure and intention-related relevance across plant communities. **(a–c)** Correlation matrices showing relationships among plant odor perception profiles within each community type. Correlation strength is indicated by color intensity and significance by asterisks. **(d–f)** Estimated marginal effects (± 95% CI) from general linear models for three perceptual dimensions: perceived intensity, emotional response, and hedonic valence.

Vegetation context was associated with changes in the correlation structure of plant-derived odor perception (**Figures 6a–c**). In the lawn setting, correlations among odor perception profiles were relatively mixed, whereas the open woodland and tree–shrub–herb contexts showed clearer species-dependent association patterns, including both positive and negative relationships among plant odor profiles. Fixed-effect models further showed that plant species had significant effects on all three semantic dimensions, namely perceived intensity, emotional response, and pleasantness (all p < 0.001). Spatial type also showed significant main effects, particularly for perceived intensity and emotional response (p < 0.001). The Plant × Space interaction was not significant for perceived intensity or pleasantness, but was significant for emotional response (p < 0.01), indicating that spatial context more strongly modulated species-specific emotional responses than intensity or pleasantness. Estimated effect sizes showed that Cp, Gj, Of, and Pm had positive effects on perceived intensity, whereas Ps showed a weaker positive effect and lawn context showed a negative effect (**Figure 6d**). For emotional response and pleasantness, most floral aromatic species showed positive effects, while Ps showed consistently negative effects (**Figures 6e, f**). Open woodland showed positive effects across semantic dimensions, whereas lawn showed weaker or negative effects. Together, these results indicate that plant identity was the primary factor differentiating odor perception, while vegetation context further modulated perceptual outcomes, especially emotional responses.

### 3.6 Odor exposure and semantic perception are associated with stop and avoid intentions

Logistic regression analysis identified different predictors of self-reported stop and avoid intentions under plant-derived odor conditions (**Table 4**). Models using stop and avoid as binary outcomes showed good internal discrimination, with AUC values of 0.862 ± 0.021 for stop intention and 0.890 ± 0.010 for avoid intention. Stop intention was associated with favorable perceptual and exposure-related predictors. Among plant species, Of showed the strongest positive association with stop intention (OR = 2.09, p = 0.026), followed by Gj, Cp, and Pm. The log-transformed dominant odor exposure metric was also positively associated with stop intention (OR = 2.20, p = 0.044), together with positive odor characteristics, perceived intensity, and pleasantness.

**Table 4:** Predictors of stop and avoid behaviors under plant-derived odor conditions.

Variable	Stop			Avoid		
	OR	95% CI	P	OR	95% CI	P
Plant species						
Cp	1.42	1.21–1.82	0.037	0.35	0.18–0.68	0.012
Gj	1.78	1.46–1.99	0.012	0.30	0.15–0.62	0.003
Of	2.09	1.60–2.38	0.026	0.67	0.53–0.85	<0.001
Pm	1.18	1.02–1.39	0.026	0.40	0.20–0.75	0.045
Ps	0.63	0.31–1.25	0.059	2.20	1.30–3.80	0.003
So	0.97	0.38–1.32	0.078	1.28	0.94–1.58	0.065
Space type						
Lawn	2.15	1.40–3.30	0.016	0.6	0.38–0.92	0.034
Open woodland	1.45	0.99–2.10	0.054	0.75	0.45–1.25	0.163
Odor characteristics						
Log dominant OAV	2.20	1.10–4.30	0.044	1.01	0.85–2.10	0.62
Odor positive	3.12	1.80–5.40	0.005	0.51	0.27–0.95	0.035
Odor negative	1.02	0.75–1.32	0.098	2.23	1.41–3.54	<0.001
Semantic perception						
Weak–Strong	3.41	2.30–5.00	<0.001	0.45	0.32–0.68	<0.001
Tense–Relaxed	1.15	0.79–1.68	0.566	0.60	0.43–0.95	0.001
Unpleasant–Pleasant	4.82	2.80–8.20	<0.001	0.78	0.61–1.00	0.047

For Plant species, CK was the reference category; for Space type, tree–shrub–herb community was the reference category. Odor positive and Odor negative were binary variables coded as 0/1, with 0 as the reference category. Log dominant OAV was log10(x + 1)-transformed. Semantic perception variables were continuous SD-scale predictors. OR, odds ratio; CI, confidence interval. The full model outputs are provided in **Supplementary Table 5**. Model discrimination performance: Stop AUC = 0.862 (5-fold CV, SD = 0.021); Avoid AUC = 0.890 (5-fold CV, SD = 0.010).

In contrast, avoid intention was mainly associated with Ps and negatively valenced odor attributes. Ps showed the strongest positive plant-species association with avoid intention (OR = 2.20, p = 0.003), whereas Cp, Gj, Of, and Pm were negatively associated with avoidance. Negative odor characteristics were strongly associated with avoid intention (OR = 2.23, p < 0.001), while positive odor characteristics and pleasantness were associated with reduced avoid intention. The log-transformed dominant odor exposure metric was not significant in the avoid model (p = 0.62), suggesting that avoidance was more closely related to odor valence than to exposure magnitude alone. Spatial context further differentiated behavioral-intention patterns, with lawn showing lower avoid intention relative to the reference context (OR = 0.60, p = 0.034). Full model estimates, 95% confidence intervals, and p values are provided in **Table 4**. Overall, these results suggest two odor-associated intention patterns: positive, intense, and pleasant odor experiences were linked to stop intention, whereas negatively valenced odor profiles were linked to avoid intention.

## 4 Discussion

### 4.1 From volatile emission to dominant odor-active signatures

Chemical profiling of the six plant species showed that floral odor profiles differed strongly among species, indicating that plant identity provides the primary chemical basis for olfactory differentiation. This result is consistent with the established view that floral scent is a species-specific signal shaped by divergent volatile biosynthetic profiles (Raguso, 2008; Schiestl, 2015). It also aligns with recent plant smellscape research emphasizing that plant-derived scents constitute an important but still under-quantified component of urban green-space experience and human well-being (Wang et al., 2025; Taufer et al., 2025; Tucker et al., 2026). However, transformation of volatile abundance into OAV-weighted profiles revealed that interspecific odor differentiation was more closely associated with a limited subset of dominant high-OAV compounds than with total volatile emission alone. This supports the key-odorant principle developed in aroma research (Grosch, 2001) and suggests that the perceptual relevance of plant odor profiles can be approximated by a sparse set of threshold-weighted compounds.

The dominant odor-active sets also varied across sampling times, indicating that temporal changes in scent perception were not simply driven by total emission intensity. Instead, species-specific shifts in the relative contributions of compounds such as indole, linalool-related monoterpenes, ionone derivatives, benzenoid esters, aromatic aldehydes, and eugenol-related phenylpropanoids altered the odor-active structure of each species. This pattern was consistent with the spatiotemporal OAV results, in which Cp and Of showed stronger source-level odor-active exposure around midday, So and Pm peaked later in the day, Ps showed a morning-dominant indole-related profile, and Gj maintained relatively high exposure in the morning and evening. These results suggest that perceptible odor events are shaped by both emission timing and the temporal reconfiguration of dominant odor-active compounds. This finding is consistent with psychophysical evidence that odor perception depends nonlinearly on concentration and is strongly influenced by detection thresholds and mixture composition (Stevens, 1957; Thomas-Danguin et al., 2014). Nevertheless, OAV should be interpreted as an exposure-based screening metric rather than a direct perceptual output, because mixture interactions, masking, adaptation, and individual semantic interpretation can modify perceived odor quality. Thus, OAV-weighted analysis is most useful for identifying candidate dominant odor-active compounds, while semantic evaluation remains necessary for determining how these chemical signals are perceived in real landscape settings.

### 4.2 Vegetation context shapes perceptible odor ranges

Distance-gradient analysis showed that odor-active exposure generally declined with distance, although attenuation magnitude differed among plant species and compound classes. Because source-level floral headspace samples were expressed as OAVs, whereas ambient air samples were expressed as relative OAV-based exposure indices, these results should be interpreted as relative source-to-ambient attenuation patterns rather than absolute concentration decay curves. Odor persistence therefore depends not only on volatile abundance, but also on compound-specific odor thresholds, source strength, atmospheric dilution, and vegetation-mediated background complexity. This interpretation is consistent with recent studies showing that floral odor signals are transmitted as complex blends in dynamic atmospheric environments and that their detectability can be modified by environmental conditions and odor-plume structure (Chan et al., 2024; Sprayberry et al., 2026). The smellwalk results further supported these exposure patterns. Species with different dominant odor-active compounds showed distinct perceptible ranges across vegetation contexts. *Photinia serratifolia*, characterized by indole-related compounds, exhibited the longest olfactory detection range, especially in the tree–shrub–herb community, consistent with the longer-distance persistence of indole-related signals. In contrast, *Prunus mume* showed the shortest detection range, matching its rapidly attenuating benzenoid ester-dominant odor profile. Other aromatic species, including *Chimonanthus praecox*, *Osmanthus fragrans*, *Gardenia jasminoides*, and *Syringa oblata*, showed intermediate perceptible ranges, reflecting the combined contributions of monoterpenes, oxygenated monoterpenes, aromatic aldehydes, ionone derivatives, esters, and phenylpropanoid compounds. Thus, smellwalk-based detection ranges provide a human-scale perceptual complement to OAV-based attenuation analysis, while remaining distinct from absolute concentration-field measurements (Parker et al., 2024).

Vegetation context further modulated these perceptible ranges. Lawn settings allowed open odor dispersion and low background interference, but odor continuity at longer distances was less stable. Open woodland provided a balance between diffusion and retention, generally supporting longer and more stable perceptible ranges for aromatic species. By contrast, the tree–shrub–herb community increased local odor retention and background complexity, reducing the clear recognition range of several pleasant floral odors but amplifying the persistence of the pungent odor of *P. serratifolia*. This context-dependent pattern supports the view that plant scents should be understood as spatially situated experiences shaped by source properties, design context, accessibility, and human perception (Lygum and Xiao, 2025). Therefore, perceptible odor boundaries should not be regarded as fixed physical distances or directly fitted diffusion parameters. Rather, they are best interpreted here as smellwalk-based detection ranges jointly shaped by odor-active chemistry, distance-dependent attenuation, vegetation structure, airflow conditions, and background odor complexity. This interpretation is consistent with atmospheric dispersion and odor-plume studies showing that outdoor odor signals are affected by wind speed, turbulence, intermittency, and spatial structure (Pasquill and Smith, 1983b; Celani et al., 2014; Riffell et al., 2014). By linking relative OAV-based index attenuation with human perceptual detection, this study provides a landscape-relevant indicator connecting chemical exposure with human-scale sensory experience, consistent with recent evidence that plant-based scentscapes are associated with human health and wellbeing in green spaces (Tucker et al., 2026).

### 4.3 Linking odor-active exposure and semantic perception to stop–avoid intentions

**Figure 7:**
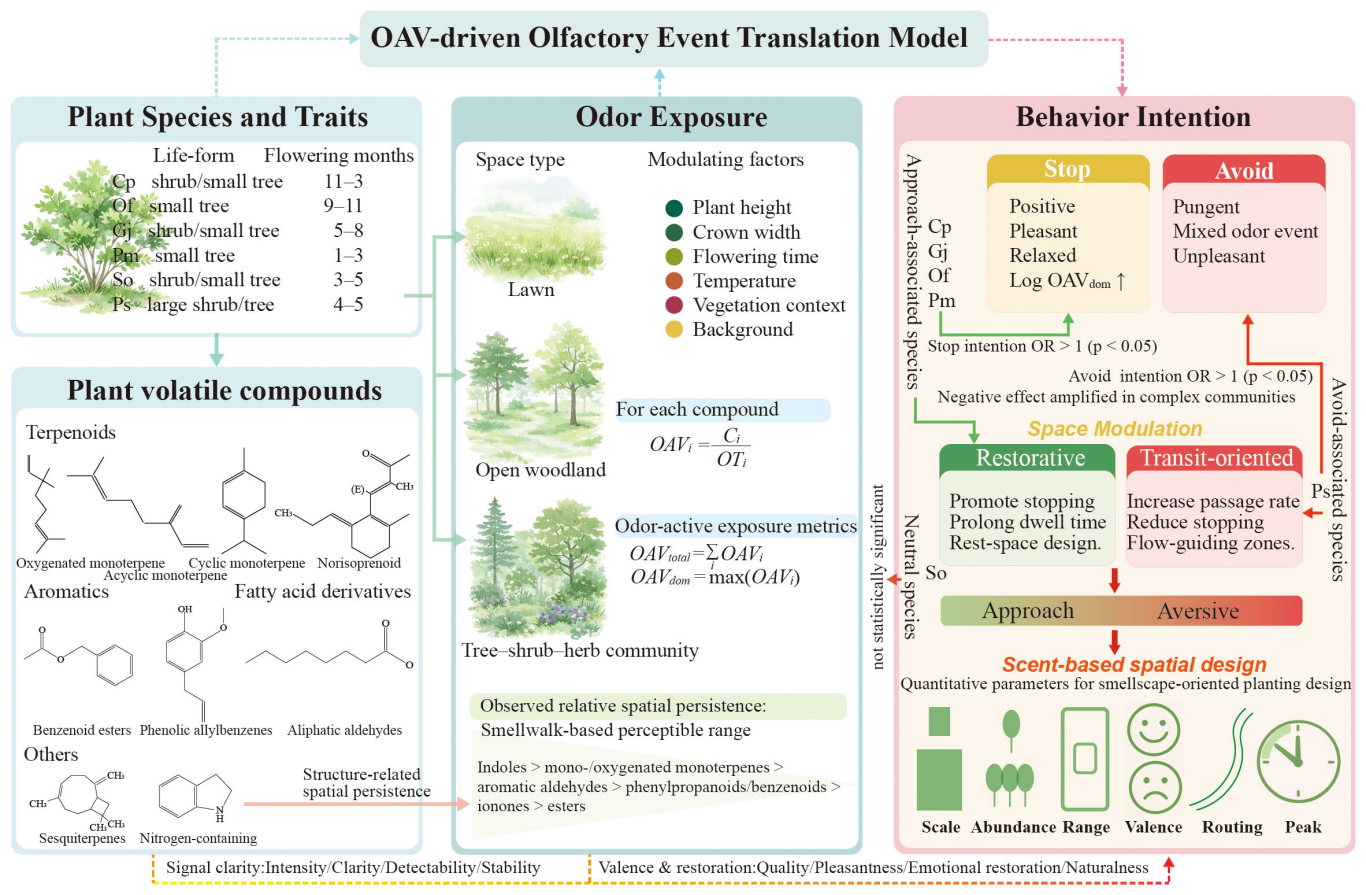
OAV-based Olfactory Landscape Translation Framework. This ranking indicates the relative persistence observed in this study, rather than an absolute physicochemical diffusion order.

Behavioral-intention modeling showed that stop and avoid intentions were associated with different components of the odor experience. Stop intention was positively associated with dominant odor-active exposure, expressed as log-transformed dominant OAV or OAV-based exposure index, and with favorable semantic attributes such as pleasantness, clarity, recognizability, and relaxation. In contrast, avoid intention was more closely linked to negative odor valence, pungency, mixed odor impressions, and vegetation-context complexity, particularly for *P. serratifolia*. These results indicate that odor intensity alone does not determine behavioral intention; instead, stop–avoid intentions were associated with the combined effects of exposure strength, semantic interpretation, and spatial context. This pattern is consistent with stimulus–emotion–behavior frameworks, in which environmental stimuli influence behavioral tendencies through affective appraisal rather than physical intensity alone (Nasar, 1994; de Groot et al., 2015). It also agrees with recent olfactory studies showing that odor valence can shape motivational and decision-related responses, with unpleasant odors more strongly associated with avoidance-related tendencies than pleasant or neutral odors (Wu et al., 2026; Yang and Wang, 2025). In this study, dominant OAV or relative OAV-based exposure indices represented odor-active signal strength, semantic dimensions captured perceptual and affective interpretation, and stop/avoid probabilities represented self-reported behavioral intentions, thereby linking chemical exposure, perceptible range, semantic meaning, and behavioral intention within a single framework (**Figure 7**). Importantly, these associations should be interpreted as probabilistic rather than deterministic, as olfactory responses emerge from the combined effects of chemical exposure, perceptual appraisal, individual experience, and spatial context (Thomas-Danguin et al., 2014; Kato et al., 2022).

Nevertheless, the behavioral component should be interpreted as intention-based rather than as direct evidence of actual movement behavior, because stop and avoid were measured as self-reported willingness to remain or leave rather than observed stopping duration, walking speed, or trajectory change. In addition, although survey timing and low-wind conditions were standardized, field odor perception may still have been influenced by short-term temporal variation in volatile emission, microclimatic fluctuation, airflow intermittency, and background odor complexity. Thus, the models indicate statistical associations rather than behavioral causality, and future studies should combine self-reported evaluations with behavioral observation, GPS-based movement tracking, and *in situ* sensory ethnography to better assess how plant-derived odors influence actual spatial behavior in urban environments. From an applied perspective, this framework provides design-relevant parameters for smellscape-oriented planting: dominant OAV or OAV-based exposure indices describe odor-active signal strength, smellwalk-based perceptible range indicates the spatial extent of odor influence, semantic dimensions characterize experiential quality, and stop–avoid probabilities provide preliminary estimates of user response tendencies. Aromatic species with stable and positively valenced odor profiles may be suitable for rest spaces, entrances, and short-stay nodes, whereas pungent or negatively valenced species with long perceptible ranges should be used cautiously near high-stay areas. Thus, the framework supports OAV-informed species selection, perceptible odor-boundary design, and intention-informed smellscape planting strategies.

## Data Availability

The original contributions presented in the study are included in the article/**Supplementary Material**, further inquiries can be directed to the corresponding author.
